# The successful implementation of the Enhanced Recovery After Surgery (ERAS) program among caesarean deliveries in Bhutan to reduce the postoperative length of hospital stay

**DOI:** 10.1186/s12884-021-04105-9

**Published:** 2021-09-18

**Authors:** Tshering Tamang, Tashi Wangchuk, Choning Zangmo, Tshering Wangmo, Karma Tshomo

**Affiliations:** 1 Department of Obstetrics and Gynaecology, Mongar Regional Referral Hospital, Mongar, Bhutan; 2 Department of Anesthesiology, Mongar Regional Referral Hospital, Mongar, Bhutan; 3 Maternity ward, Department of Obstetrics and Gynaecology, Mongar Regional Referral Hospital, Mongar, Bhutan

**Keywords:** Caesarean deliveries, ERAS, Length of hospital stay

## Abstract

**Background:**

Enhanced Recovery After Surgery (ERAS) is a multidisciplinary perioperative care program to optimize and enhance postoperative recovery. It has a beneficial role in decreasing the length of hospital stay and improving the quality of care. This study aims to observe the successful implementation of ERAS in reducing the length of hospital stay (LOS) among caesarean deliveries.

**Methods:**

A pre-and post-implementation study of ERAS protocol was conducted, among cohort of women who underwent caesarean deliveries from January to December 2020 in the Department of Obstetrics and Gynaecology, Mongar Regional Referral hospital. Data collected retrospectively and analyzed in SPSS (IBM SPSS trial version); and comparison of length of hospital stay between the two groups were tested by t-test.

**Results:**

One hundred seventy-one patients were included in the study: 87 in the pre-ERAS and 84 in the post-ERAS cohort. Post implementation, LOS decreased by an average of 21.0 (CI 16.11–24.64; *p*-value < 0.001) hours in the postoperative period. A greater proportion of patients were discharged on day-2 (2.3% in pre-ERAS and 81% in ERAS; *p*-value < 0.001).

**Conclusion:**

Implementation of ERAS protocol can significantly decrease the postoperative length of hospital stay without increasing the complications and readmission rates.

**Supplementary Information:**

The online version contains supplementary material available at 10.1186/s12884-021-04105-9.

## Background

Enhanced Recovery After Surgery (ERAS) is a multidisciplinary perioperative care program that combines evidence-based practices to optimize and enhance patient recovery. The goal of the ERAS pathway is to reduce surgical stress and accelerate early physiological and functional recovery in the postoperative period. Its role in decreasing the length of hospital stay, potential complications, readmission rates, and financial burden to the healthcare system have been documented [1–4]. The ERAS Society (www.erassociety.org) is an international, multidisciplinary, non-profit organization that has developed guidelines and made recommendations for all surgical disciplines [5]. The society recommends to focus on a patient-centric approach and utilize specific elements of ERAS during Caesarean deliveries (CD) in the perioperative period for improved enhancement of the maternal and fetal health outcomes [6–8].

Successful implementation of ERAS protocol among CD require a concerted multidisciplinary team approach and standardization of care to improve quality of patient care [9, 10]. In many of our hospitals, the utilization of the ERAS pathway in perioperative care has remained a new concept; and has not been implemented in different surgical fields. With the adoption of the ERAS program in our department, we aimed to describe the successful implementation of ERAS protocol with a primary outcome to compare the length of post-operative length hospital stay (LOS) in ERAS and the traditional care among Caesarean deliveries. A secondary aim was to determine surgical complications in the ERAS pathway.

## Methods

### Study design, setting, and participants

After prior protocol approval from Research Ethics Board, Ministry of Health (Ref.No.REBH/Approval/2021/041), we designed a pre- and post-ERAS implementation study retrospectively to include cohort of women who underwent CD in the department of Obstetrics and Gynaecology, Mongar Regional Referral Hospital. The facility department experiences about 800 hundred deliveries annually with caesarean rate of 34.4% [11]. The patient records who underwent CD in 2020 were collected from medical record department CD; reviewed and recruited for this study; irrespective of its indications, type (emergency or elective), comorbidities (presence or absence), and complications. Exclusion criteria included women who had prolonged stay due to other reasons like preterm delivery, social and financial incapacities, and inability to follow up. Recruited patients delivered by cesarean section from January to June, 2020 were on pre-ERAS care; and those from July to December, 2020 on post-ERAS program. Data variables included patient’s basic demographic and obstetric details, Body Mass Index (BMI), associated medical comorbidities, indication for caesarean, postoperative LOS in hours, and surgical complications particularly surgical site infection experienced by the patient from immediate post-operative period to 30 days follow up.

### Pre-ERAS perioperative care

Traditionally, the patients scheduled for elective CD were admitted a day prior, and a strict fasting period was ordered from midnight till the time of surgery. Preoperative patient education and counseling were limited, and medications and fluid loading were inconsistently practiced. Preoperative oral antibiotics and vaginal cleansing with an antimicrobial solution for emergency CD were not routine. The skin was prepared with povidone-iodine alone. Surgical techniques and skin closure depended upon the experience and expertise of the surgeon. Interventions to prevent hypotension and hypothermia were not routinely a part of the preoperative procedures, and intravenous fluids were administered liberally. All patients had prolonged fasting, prolonged immobilization, and longer urinary catheter placement after surgery along with intramuscular analgesics. Women were discharged on the third day in stable clinical condition, and then followed-up in the postnatal clinic.

### ERAS protocol implementation

The ERAS protocol was implemented from 1st July 2020 for all gynecological procedures and CD in the department. Prior to implementation, education on ERAS care was conducted for the care-providers from maternity ward, operation theatre, and hospital quality assurance division in a common presentation. The focus of this presentation stressed on the processes and elements of ERAS, individual-specific roles and the standardization of care during the perioperative period. While the operating surgeon and the anesthesiologist played the central role in its implementation, the nursing staffs and technicians in the ward and operating room had executed crucial roles to carry out the elements of ERAS. The hospital quality assurance division was involved from the conception stage to avoid compromise in the quality of care. This was followed by development of Standard Operation Procedure (SoP) in accordance to the guidelines and the recommendations developed by the ERAS society [6–8, 12]. The document was distributed to the ward and operation theatre for consistent and independent use by the nursing and technical personnel. For each patient, a checklist on the specific components was instituted for the staffs to ensure provision of particular care. Difficulties in the implementation and executing the components were dealt at individual level, and corrective actions and revision of the SoP was done at intervals. The implementation period was initiated from 1st July to 31st December, 2020.

### ERAS perioperative care

In the ERAS standard of care, all parturient women were provided with information on risks, benefits, and complications of the caesarean deliveries; pain management plan; goals for early feeding, and mobilization were conveyed. Patients were given the option of admission on the day of surgery for a scheduled CD. The practices adopted were from the evidence-based ERAS protocol particularly on the preoperative fasting and medication, intraoperative skin preparation, postoperative early feeding, early mobilization, early catheter removal. These practices were uniformly applied to both emergency and elective CD. Table 1 elaborates the changing practices in the pre- and post-ERAS protocol among CD patients care. Patients who were clinically stable, satisfactorily ambulating, with adequate oral intake and acceptable pain control on oral analgesics were discharged on day 2 of surgery. Any patient with medical condition needing further investigation and care was not discharged until stable. Follow-up was done in the post natal visits in the clinic at 7, and 21 days; and telephonically at 30 days.

**Table 1:** Perioperative management in pre- and post-ERAS program in Caesarean deliveries practiced in Mongar Regional Referral hospital

Components	Pre-ERAS	Post-ERAS
Pre-operative		
• Fasting	Overnight	6 h for solid and 2 h for clear liquid
• Medication	IV Ranitidine; IV Metoclopromide	Oral Ranitidine and Antacid; Acetaminophen 1 g 2 h prior
• Antibiobiotic prophylaxis	IV Cephazolin 2 g within 1 h	IV Cephazolin 2 g; Oral Azithromycin 1 g for laboring and/or membrane ruptured patients
• Skin preparation	Povidone iodine alone	Alcohol-based Chlorohexidine or Iodine-Alcohol mixed-preparation
• Vaginal preparation	None	Povidone iodine for all emergency cases
Intraoperative		
• Anaesthesia	Neuraxial	Neuraxial
• Analgesia	None	LA infiltration of wound (subcutaneous) and IM PCM 600 mg
• Hypothermia prevention	None	Forced air/ Temperature monitoring
• Surgical procedure	Blunt technique Skin – Interrupted suture	Blunt technique Re-approximation of subcutaneous layer if thickness > 2 cm Skin – Subcuticular suture
• Fluid management	Liberal ~ 4 to 5 l first 24 h	Goal directed, Early stopping of IV fluids
• New born care	Delayed cord cutting and early essential neonatal care not part of routine care	Routinely done
Postoperative		
• Analgesia	IM Pethidine and IM Diclofenac	Intermittent IV Morphine for 24 h; Combination of Oral PCM and Ibuprofen started from 3 h
• Feeding and parental fluid restricting	Fasting for solid and liquid for 24 h	Fasting for 3 h; Limited IV fluid Normal diet as early as 6 h
• Mobilization	Not done until next day	Routinely done after 6 h Physiotherapy from day 1
• Catheter removal	Removed in postoperative day 1	Routinely removed after 6 h

### Study outcomes

The primary outcome for our study was the postoperative length of hospital stay (LOS) calculated in hours from the time of surgery till discharge for all cases of CD before or after the implementation of ERAS. We also looked at the surgical complication rates following surgery particularly surgical site infection and readmission due to any cause.

### Statistical analysis

The data management and validation was done using Epidata (version 3.1) and statistical analysis was done using SPSS (IBM SPSS Trial version). Obstetrics variables were analyzed using descriptive tests like proportion and percentages. Continuous variables were compared between the two groups by t-test and categorical variables by chi-square test. *p*-value < 0.05 and 95% CI was used to calculate the level of significance.

## Results

A total of 176 women underwent CD during the year 2020, 91 in pre-ERAS and 85 in post ERAS arm. Four women in pre-ERAS and one in the post-ERAS group were excluded after confirming their prolonged stay due to preterm delivery, financial and social reasons. Eighty-seven women before and 84 after ERAS introduction were eligible for analysis. In both the groups, homogeneous distribution was observed in demographic, preoperative characteristics, CD, and its indications. The mean age in the pre-ERAS (29.2 ± 6.1 years) and post-ERAS (29.6 ± 6.3 years) group were not statistically significant. The mean parity was also not significant statistically. The proportion of co-morbid conditions like hypertensive disorders (10.3% vs 14.3%), GDM (2.3% vs 2.4%), anemia (5.7% vs 9.5%) and obesity (17.2% vs 19.0%) were slightly higher in post-ERAS care. More elective CDs were done in both the groups (55.2 and 56.0%). Previous Caesarean was the commonest indication for CD (42.5 and 44.0%). The difference in postoperative-hemoglobin level between the two groups was not significant (*p*-value 0.22); only small numbers received blood transfusion (3.4 and 4.7%). Table 2 describes the demographic and clinical characteristics of women who underwent CD in 2020.

**Table 2:** Demographic and clinical characteristics of women who underwent CD in 2020 in Mongar Regional Referral hospital

Demography	Pre-ERAS (*n* = 87)	Post-ERAS care (*n* = 84)	Significance “*p*” value
Mean Age	29.3 ± 6.1	29.7 ± 6.3	0.68
Parity			
0–1	58 (66.7%)	51 (60.7%)	0.51
≥ 2	29 (33.3%)	33 (39.3%)	
Co-morbidities			
Hypertension	9 (10.3%)	12 (14.3%)	0.76
GDM	2 (2.3%)	2 (2.4%)	1.0
Anemia	5 (5.7%)	9 (10.7%)	0.56
Obesity	15 (17.2%)	13 (15.5%)	0.27
Caesarean			
Emergency	39 (44.8%)	37 (44.0%)	
Elective	48 (55.2%)	47 (56.0%)	
Indications			
Past CS	37 (42.5%)	37 (44.0%)	0.84
Fetal indications	28 (32.2%)	23 (27.4%)	0.49
Maternal indications	7 (8.0%)	9 (10.7%)	0.54
Labour dystocia	6 (6.9%)	9 (10.7%)	0.38
Others	9 (10.3%)	6 (7.1%)	0.46
Pre-op hemoglobin	12.6 ± 1.1	11.9 ± 1.3	0.00
Post-op hemoglobin	10.5 ± 1.2	10.3 ± 1.3	0.33
Blood transfusion	2 (2.3%)	4 (4.8%)	0.44

After ERAS implementation, the postoperative length of stay was significantly lower than in pre-ERAS care. The mean time was reduced by 21.0 h (CI 16.11–24.64; *p*-value < 0.001) in the postoperative period. A higher proportion of patients were discharged on day 2 in post-ERAS arm (82.1%) compared to pre-ERAS (3.2%) arm (*p*-value < 0.001). The differences were also observed in emergency and elective CD. In the pre- and post ERAS implementation the mean LOS in emergency and elective reduced by 23 and 19.3 h respectively, which were independently significant. A greater proportion of patients were discharged on day-2 (2.3% in pre-ERAS and 81% in ERAS; *p*-value < 0.001).

Thirty days follow-up after surgery revealed, surgical site infection was the main complication for readmission in both groups. Three (3.4%) women in pre-ERAS arm and two (2.4%) in post-ERAS arm required debridement and resuturing in the operation theatre. The anesthetic complications included nausea and vomiting, spinal headache. Table 3 depicts the LOS and complications experienced by the women in two groups.

**Table 3:** Postoperative length of stay and complications experienced by in the pre-and-post ERAS groups

	Pre-ERAS (*n* = 87)	Post ERAS (*n* = 84)	Significance (*p* value)
Postoperative length of stay (hours)			
Overall	72.7 ± 13.4	51.7 ± 15.4	0.00
Range	48–175	24–116	
•Emergency CD	74.3 ± 19.3	51.3 ± 17.2	0.00
•Elective CD	71.4 ± 5.0	52.1 ± 14.0	0.00
Discharges			
Day 2	2 (2.3%)	68 (81.0%)	0.00
Complications			
SSI	3 (3.4%)	2 (2.4%)	0.63
Readmission	3 (3.4%)	3 (3.6%)	1.0
Postop nausea & vomiting	2 (2.3%)	2 (2.4%)	0.63
Post spinal headache	10 (11.5%)	9 (10.7%)	0.66
UTI	1 (1.1%)	1 (1.2%)	1.0

## Discussions

The main intent of implementing the ERAS program was to promote patients’ early return to mobility and function, to reduce the length of stay in the hospital, and to decrease rates of post-surgical complications. Our study demonstrated a statistically significant reduced postoperative length of stay by 21.0 h after ERAS implementation compared to the traditional care without increasing the complication and readmission rates. The findings of shorter length were consistent with the study done by Mullman et al., who was able to demonstrate a reduction of LOS by 0.8 days [13]. Due to shorter stay, a decrease in the hospital associated costs has been observed along with the reduction in administration of opioids in the postoperative period [2, 14]. This implicates a reduction in expenditure for free healthcare like ours in Bhutan where the sole funding comes from the state.

Most of the studies on ERAS have been done in high-income countries and among elective CD where planning and application of all elements of ERAS were possible. Among emergency CD, all components of pre-operative care are not applicable, unlike a planned CD. The difference in LOS and complication rates in both elective and emergency CD was not significant as observed in our data. The LOS in our emergency CD was similar to the randomized controlled trial conducted in one of the African referral hospitals which demonstrated a reduction by 18.5 h in LOS in emergency CD after ERAS implementation [15]. Early oral intake, early ambulation, and early catheter removal in the postoperative period might have played important role in faster recovery.

An early discharge from the hospital is one of the hallmarks of ERAS pathway adoption. In our study, day 2 hospital discharges after the implementation was significantly and proportionately higher (81% vs 2.3%); which was as opposed to the pre-ERAS where greater day 3 discharges were observed (88.8%). A study by Wrench et.al in their tertiary center also concluded that a greater proportion of patients discharged on Day 1 can be achieved after the ERAS program without compromising the quality [16].

Before the introduction of the ERAS protocol, the practice of perioperative care in our department was not standardized and heavily dependent on the operating surgeon. The success of our protocol was driven by the multidisciplinary approach of the ERAS program which provided a standard procedure of peripartum care for all parturient mothers. It is noteworthy to explain that, compliance to the protocol guidelines was strictly adhered by the operating surgeon, nursing staffs, and anesthesia team. Much of the success is attributed to the collaborative care imparted by the nursing team. Although it appears to increase the workload of nursing staff to adhere to many elements of ERAS care, the overall load is substantially lower due to shorter hospital stay [17].

There is growing evidence that ERAS protocol in CD is safe, feasible, and effective. The newer shreds of evidence and practices have been published by ERAS society in three parts [6–8]. Recent consensus and recommendation had been made by Society for Obstetric Anesthesia and Perinatology [18]. Our protocol development was in uniformity with these guidelines. Some of the positive changes observed in our ward were; avoiding prolonged fasting,limiting parental fluid and encouraging oral intake as early as in 3 h post operatively, assisting to mobilize after 6 h, removing urinary catheter at 6 h, practicing early essential newborn care (skin to skin contact); and discharging the mother home early. Implementing the ERAS protocol is not difficult and it is associated with several improved maternal and fetal outcomes which are well documented and conveyed by earlier studies [13, 19].

This study is not without limitation. It only reveals the postoperative length of stay of the mother. It does not dwell on the total duration of stay in the hospital, because some mothers tend to stay pre-operatively due to financial reasons and distance from the hospital. The discharge criteria were formulated for the mothers only. Some babies are admitted for the continuation of care which prolongs their stay. The study does not describe this in the exclusion criterion. It was also difficult to demonstrate which individual element of ERAS had the profound effect. However, this study has portrayed the success of the implementation of the ERAS pathway which forms a basis for future implementation in other hospitals and surgical specialties.

## Conclusion

Implementation of ERAS protocol can significantly decrease the postoperative length of hospital stay without increasing the complications and readmission rates. Such quality improvement initiatives and evidence-based practices need to be adopted in our surgical practices to improve the peripartum care of the mothers and the babies. It is additionally more significant due to its success in a young nation like Bhutan.

## Supplementary Information

**Additional file 1.**

## Data Availability

The datasets used and/or analysed during the current study available from the corresponding author on reasonable request. All data generated or analysed during this study are included in this published article [and its supplementary information files].
